# Diagnostic Performance of Gynecologic Imaging Reporting and Data System (GI-RADS) in Preoperative Evaluation of Adnexal Masses

**DOI:** 10.3390/medicina61040679

**Published:** 2025-04-07

**Authors:** Ahmed A. Taha, Sara Abdallah Mohamed Salem, Eman Zein El Abdeen Faried, Eman Hosni Habib, Reham S. Al-Fakharany, Marwa O. Elgendy, Hamdy Abdelkader, Adel Al Fatease, Maged Salah eldien Elkady

**Affiliations:** 1 Obstetrics & Gynecology Department, Faculty of Medicine, Beni-Suef University, Beni Suef 62521, Egypt; ahmedabdulkhaliq@med.bsu.edu.eg (A.A.T.);; 2 Obstetrics and Gynecology Department, Fayoum General Hospital, Fayoum 63513, Egypt; emanhosny1611@gmail.com; 3 Physiology Department, Faculty of Medicine, Beni-Suef University, Beni Suef 62521, Egypt; drrehamelfakharany@gmail.com; 4 Department of Clinical Pharmacy, Beni-Suef University Hospitals, Faculty of Medicine, Beni-Suef University, Beni Suef 62521, Egypt; 5 Department of Clinical Pharmacy, Faculty of Pharmacy, Nahda University (NUB), Beni Suef 19206, Egypt; 6 Department of Pharmaceutics, College of Pharmacy, King Khalid University, Abha 62529, Saudi Arabia; habdelkader@kku.edu.sa (H.A.); afatease@kku.edu.sa (A.A.F.); 7 Obstetrics & Gynecology Department, Faculty of Medicine, Misr University for Science and Technology, Cairo 12566, Egypt; magedelkady@hotmail.com

**Keywords:** gynecologic imaging, reporting and data system, adnexal masses, ovarian cancer

## Abstract

*Background and Objectives:* Ovarian cancer is a highly lethal gynecological malignancy and the fifth leading cause of cancer-related deaths. Diagnosis mainly involves gynecological examination and transvaginal ultrasonography. To evaluate the diagnostic performance of the Gynecology Imaging Reporting and Data System (GI-RADS) with regard to its ability to evaluate adnexal masses preoperatively, considering a definitive histopathological diagnosis. *Materials and Methods:* This study was approved by the ethics committee, and informed consent was obtained from all the patients. This research was conducted at Beni-suef University Hospital between June 2021 and January 2023 on 100 women who experienced pelvic pain due to an adnexal mass. *Results:* Our study results revealed that the combination of IV-V GI-RADS had high specificity (92.2%), sensitivity (87%), and a negative predictive value (95.9%), but moderate other diagnostic characteristics for predicting adnexal mass malignancy. *Conclusions:* The GI-RADS classification system is a reliable method for reporting ovarian masses, with high diagnostic accuracy for predicting malignancy. It aids in patient triage and clinical decision making. To optimize care, it is essential to inform referring clinicians about the objectives of the GI-RADS before its implementation in a treatment plan.

## 1. Introduction

Ovarian cancer is an aggressive and lethal disease, ranking as the highest cause of mortality among gynecological cancers [1]. Accurately distinguishing between benign and malignant adnexal masses during the preoperative stage, especially those with complex ultrasound features, remains a critical challenge for sonographers [2]. The standard treatment for ovarian cancer typically involves extensive debulking surgery followed by platinum-based chemotherapy [3]. Conversely, benign tumors are often managed through fertility-preserving surgery or regular monitoring [4]. Hence, a comprehensive preoperative assessment is essential for the effective management of ovarian cancer [5]. Ultrasonography (US) serves as the primary imaging technique for diagnosing and differentiating adnexal masses [6]. In 2009, Amor et al. [7] introduced the Gynecology Imaging Reporting and Data System (GI-RADS), a structured assessment framework akin to the Breast Imaging Reporting and Data System (BI-RADS) used in breast imaging. GI-RADS aims to improve communication between radiologists and clinicians about the likelihood of malignancy, facilitating better treatment planning and outcomes [8]. The GI-RADS is a reporting system specifically designed for the documentation of findings in adnexal masses using transvaginal ultrasonography. The study categorizes the results into five categories: in GI-RADS 1, typical ovaries with no adnexal mass are identified. GI-RADS 2 includes functional adnexal lesions like follicles, corpus luteum, and hemorrhagic cysts. GI-RADS 3 encompasses benign neoplastic adnexal lesions such as endometriomas, teratomas, simple cysts, hydrosalpinx, peritoneal pseudocysts, pedunculated myomas, and signs indicative of pelvic inflammatory disease. GI-RADS 4 covers any adnexal lesions not mentioned in the previous categories, and the lesion exhibits one or two features suggestive of malignancy like thick papillary projections, thick septations, solid areas, ascites, vascularization within solid areas, papillary projections, and a central area of a solid tumor visualized on color or power Doppler. GI-RADS 5 includes adnexal masses with three or more features suggestive of malignancy [9].

Several studies have validated the utility of GI-RADS for adnexal mass evaluation. For example, Basha et al. (2019) reported high specificity and inter-reviewer agreement when applying GI-RADS to ultrasound findings [10]. Similarly, Khalaf et al. (2019) highlighted the diagnostic accuracy and agreement of GI-RADS categories with histopathological outcomes [11]. Despite these promising results, the system remains limited by its reliance on subjective sonographer evaluations, which can introduce variability in diagnostic accuracy [9,12].

This study aimed to investigate the diagnostic accuracy of GI-RADS in the preoperative evaluation of adnexal masses, considering histopathological confirmation as the reference standard. Additionally, it seeks to address gaps in the current literature by exploring error patterns and assessing operator dependency

## 2. Materials and Methods

A prospective study was performed with 100 women who had pelvic pain due to an accidently discovered adnexal mass attending Beni-suef University Hospital, beginning from June 2021 to January 2023. Sample size estimation was performed using G*Power (version 3.1), assuming a moderate effect size of 0.5, a power of 80%, and a significance level of 0.05. The effect size was derived from prior studies evaluating the diagnostic accuracy of GI-RADS. This ensured the sufficient representation of GI-RADS categories for robust statistical analysis.

### 2.1. Exclusion Criteria and Patient Selection

We excluded pregnant women that presented with pelvic masses and patients that showed sure signs of malignancy, e.g., cachexia pelvic lymphadenopathy, or signs of metastasis.

For all female participants, comprehensive history taking, physical examinations, and preoperative routine laboratory tests, including tumor markers, were conducted. Written informed consent was obtained after providing detailed study information. Participant privacy was strictly maintained. The data collection process received approval from the Institutional Research Ethics Committee of the Faculty of Medicine, Beni-Suef University (FMBSUREC/06072021/Habib), and complied with the Declaration of Helsinki principles.

### 2.2. Imaging Protocols

One hundred patients underwent trans-vaginal ultrasound with an endo-vaginal transducer in the lithotomy position and trans-abdominal ultrasound in the supine position in both transverse and longitudinal planes using a GE Logic P6 ultrasound. B-mode imaging, color, and spectral Doppler were utilized.

Two expert examiners with more than 10 years’ experience in gynecological ultrasound, performed all examinations. Lesions were analyzed using the GI-RADS classification system and assigned a suggested management protocol based on malignancy risk. Surgical referral and treatment planning were decided in consultation with a multidisciplinary team meeting (MDT). For GI-RADS 4 and 5 patients, definitive histopathological diagnosis was obtained through laparoscopic or surgical excision. In some GI-RADS 3 cases, a definitive diagnosis was also established.

A sensitivity analysis was performed. The specificity and negative predictive value (NPV) were calculated under a worst-case scenario assumption, where all unconfirmed cases were considered false negatives. This sensitivity analysis allows for a more accurate estimation of the true negative rate in the absence of histopathology for all cases.

### 2.3. Statistical Analysis

Data were analyzed using IBM SPSS Statistics (version 22.0), a statistical package for social sciences. Descriptive statistics, such as range (minimum and maximum values) and mean ± standard deviation were employed for quantitative data, while counts and percentages were reported for qualitative data. Inferential analyses included the Shapiro–Wilk test for normality and the independent t-test for normally distributed data in two independent groups. The Chi-square test analyzed differences in proportions for qualitative data, with Fisher’s exact test applied for small, expected numbers. Significance was set at *p* < 0.050.

## 3. Results

A total of 100 patients were recruited and their clinical data were analyzed, showing a Mean ± SD of Age (years) and BMI (kg/m^2^) at 46.4 ± 10.3 and 27.1 ± 2.3, respectively. The majority of cases were multiparous (89.0%). More than two thirds were premenopausal (69.0%). A family history of ovarian and breast cancer occurred in 5% and 8% of patients, respectively. A past history of breast cancer was present in 3.0% of cases. The most frequent GI-RADs grade was III (65.0%), followed by IV (20.0%), then II (9.0%) and V (6.0%).

The histopathology findings were reported as follows: serous cystadenoma was the most reported with at 24%, followed by endometrioma (19%), dermoid (14%), and then serous cystadenocarcinoma (16%), mucinous cystadenoma (9%), fibroma (7%), functional cysts (4%), immature teratoma (4%), and finally, mucinous cystadenocarcinoma (3%). Malignancy was found in less than a quarter of cases (23%), while benign was found in 77%.

In Table 1, Age and BMI are clearly higher in cases with malignancy. Furthermore, being multiparous, being postmenopausal, a family history of ovarian cancer, a family history of breast cancer, and a past history of breast cancer were more commonly reported in cases of malignancy. However, only having a postmenopausal status showed a statistically significant difference.

GI-RADS grades IV and V were significantly more frequent in cases with malignancy, as mentioned in Table 2.

Table 3 GI-RADS grades were significantly different among histopathology findings.

There was a substantial level of agreement between the GI-RADS diagnosis and the final histopathology diagnosis, as evidenced in Table 4.

Table 5 showed that the dermoid had the highest false positive findings (50.0%), followed by serous cystadenoma (33.3%), then endometrioma (16.7%). Serous cystadenocarcinoma was the most frequent false negative findings, followed by mucinous cystadenocarcinoma (33.3%), see Supplementary Materials.

GI-RADS revealed high specificity and negative predictive value, but moderate levels of other diagnostic characteristics in Table 6.

### 3.1. Inter- and Intra-Observer Variability

The kappa statistics demonstrated substantial agreement among sonographers (κ = 0.80, 95% CI: 0.72–0.88), indicating a good reproducibility of GI-RADS classifications. This supports the reliability of the diagnostic system when applied by trained operators.

### 3.2. Error Patterns and Clinical Implications

An analysis of false positives revealed that dermoid cysts accounted for 50% of misclassified cases, followed by serous cystadenomas (33.3%). Among false negatives, serous cystadenocarcinomas were the most frequent (66.7%). These findings underscore the need for complementary diagnostic modalities, such as biomarkers or advanced imaging techniques.

The results of specificity and NPV calculations incorporating the sensitivity analysis indicate that while the diagnostic accuracy of GI-RADS is high, the absence of histopathological confirmation in certain cases introduces a potential source of overestimation in specificity.

## 4. Discussion

Adnexal masses are a common clinical concern due to their diverse underlying causes [12,13]. Imaging plays a pivotal role in their diagnosis and classification [14], with pelvic ultrasound serving as the primary method for detecting and evaluating these masses [1]. Accurately identifying the nature of an adnexal mass is essential for determining the most appropriate management strategy [15].

However, diagnosing adnexal masses can be challenging and may yield misleading results [16]. To address this, the diagnostic performance of the Gynecologic Imaging Reporting and Data System (GI-RADS) in detecting adnexal masses via pelvic ultrasound (US) has become a key focus of investigation [10]. This study aimed to evaluate the effectiveness of GI-RADS in the preoperative malignancy assessment of adnexal masses and compare its diagnostic accuracy with the final histopathological findings.

The findings of the current study showed that the GI-RADS grade III was the most common classification (65.0%), followed by grade IV (20.0%), grade II (9.0%), and grade V (6.0%). Histopathological analysis confirmed a malignancy rate of 23% and a benign rate of 77%. The most frequently observed histopathological diagnosis was serous cystadenoma (20%), followed by endometrioma (19.0%) and serous cystadenocarcinoma (16%). These results are consistent with previous research.

A prospective cross-sectional study by Hamed [17], which included 100 women with 112 adnexal masses, evaluated the diagnostic accuracy of the GI-RADS classification system. In this study, 36 lesions (32.1%) were categorized as GI-RADS 2, 32 (28.6%) as GI-RADS 3, 13 (11.6%) as GI-RADS 4, and 31 (27.7%) as GI-RADS 5. Histopathological examination revealed a malignancy prevalence of 29.5% (33/112).

Basha et al. [10] conducted a prospective multicenter study involving 308 women with 325 adnexal masses (AMs) to evaluate the diagnostic accuracy and inter-reviewer consistency of the GI-RADS system using pelvic ultrasound.

The study identified 127 malignant (39.1%) and 198 benign (60.9%) AMs. Among the benign AMs, follicular cysts were the most frequent (18%), while serous cystadenocarcinomas were the most common malignant AMs (18.6%). The findings revealed a strong correlation between GI-RADS classifications and final histopathological diagnoses, with a kappa value of 0.757 (*p* < 0.001). These results are consistent with prior studies by Basha et al. [10], which classified 325 AMs as follows: 29.2% as GI-RADS 2 (none were malignant), 28.9% as GI-RADS 3 (three malignant), 4% as GI-RADS 4 (six malignant), and 37.8% as GI-RADS 5 (118 malignant).

Additionally, Khalaf et al. [11] also reported a high level of agreement between GI-RADS and final diagnoses, with a kappa value of 0.91. According to these studies, dermoid cysts were the most commonly misclassified as true positives (50%), followed by serous cystadenomas (33.3%) and endometriomas (16.7%). On the other hand, serous cystadenocarcinomas were the most frequent false negatives, followed by mucinous cystadenocarcinomas (33.3%).

The findings of the present study suggest that the combined GI-RADS IV-V categories demonstrated high specificity (92.2%), sensitivity (87%), and negative predictive value (95.9%) for predicting malignancy in adnexal masses. However, the diagnostic performance in other predictive aspects was moderate. These results align with those of Hamed [17], who reported an overall diagnostic accuracy for the GI-RADS classification system with sensitivity of 97%, specificity of 84.8%, a positive predictive value (PPV) of 72.7%, a negative predictive value (NPV) of 98.5%, and overall accuracy of 88.4%.

In contrast, Migda et al. [8] observed low sensitivity (66.0%) and high specificity (93.8%) for GI-RADS when combined with the CA-125 biomarker. However, GI-RADS categories 4 and 5 alone exhibited higher sensitivity (94.3%) and lower specificity (72.2%). This discrepancy may be due to differences in the number of lesions analyzed across studies. Utilizing well-defined GI-RADS sonographic criteria for evaluating adnexal masses reduces the number of inconclusive ultrasound results and limits the need for additional imaging with alternative modalities.

The diagnostic accuracy of GI-RADS aligns with that of alternative systems like the IOTA Simple Rules, which also demonstrate high sensitivity and specificity. However, GI-RADS offers the advantage of a structured, widely applicable framework. The inclusion of biomarkers such as CA-125 and HE4 in future studies could further improve diagnostic performance [18].

While the study highlights the high specificity and sensitivity of GI-RADS, its operator dependency remains a limitation. Training and standardized imaging protocols are essential to mitigate variability. Future research could explore AI-assisted tools to enhance objectivity.

This study’s merits lie in its prospective design, which eliminates selection bias and ensures no patients were lost to follow-up during the study period. Additionally, the implementation of the GI-RADS reporting system will greatly enhance communication between radiologists and gynecologists, leading to improved diagnosis and effective management of patients with adnexal lesions based on both clinical and ultrasound morphological characteristics of the lesion. The study’s limitations should be acknowledged; the GI-RADS classification solely relies on the US, which is an operator-dependent modality and should only be performed by highly experienced sonographers, as this impacts diagnostic performance. Additionally, magnetic resonance imaging (MRI) was not used in our study to compare its diagnostic accuracy with transvaginal ultrasonography (US) in evaluating GI-RADS.

The decision to exclude MRI or CT imaging reflects the study’s focus on evaluating the cost-effectiveness and accessibility of ultrasound. However, this limits the generalizability of findings. Multi-modal approaches integrating ultrasound, MRI, and biomarkers are recommended for future studies

### Limitations and Future Directions

This study’s single-center design and the exclusion of specific patient groups, such as pregnant women, limit its generalizability. Expanding future research to include multiple centers and a more diverse population would enhance the validity of the findings. Furthermore, a thorough analysis of GI-RADS II and III cases with histopathological confirmation is crucial for accurately assessing its negative predictive value.

We recognize that the absence of universal histopathologic confirmation may impact the reliability of GI-RADS in this study. While the kappa statistic indicated strong agreement among sonographers, it does not replace definitive pathological validation. To improve accuracy and reduce potential biases in specificity and true negative rates, future studies should mandate histopathological correlation for all GI-RADS 3 and 4 cases.

## 5. Conclusions

The GIRADS classification system exhibits high reliability for diagnosing AMs via US and serves as an effective and valid reporting system for ovarian masses, with excellent diagnostic performance in predicting malignancy. It is a valuable tool for patient management and clinical decision making. Explaining the purpose of the GIRADS system to referring clinicians before treatment can enhance patient care. The findings of this study can be utilized to guide future research involving larger sample sizes and to validate our outcomes against MRI.

## Figures and Tables

**Table 1:** Comparison according to final diagnosis regarding demographic characteristics.

Characteristics		Malignant (Total = 23)	Benign (Total = 77)	*p*-Value
Age (years)		51.3 ± 7.3	45.0 ± 10.6	^ 0.002 *
BMI (kg/m^2^)		28.0 ± 2.5	26.9 ± 2.1	^ 0.037 *
Parity	Nulliparous	2 (8.7%)	9 (11.7%)	§ 0.999
	Multiparous	21 (91.3%)	68 (88.3%)	
Menstruation	Premenopausal	11 (47.8%)	58 (75.3%)	# 0.012 *
	Postmenopausal	12 (52.2%)	19 (24.7%)	
Family history of ovarian cancer		3 (13.0%)	2 (2.6%)	§ 0.078
Family history of breast cancer		3 (13.0%)	5 (6.5%)	§ 0.380
Past history of breast cancer		2 (8.7%)	1 (1.3%)	§ 0.131

^ Independent *t*-test. # Chi-square test. § Fisher’s exact test. * Refers to significance.

**Table 2:** Comparison according to final diagnosis regarding GI-RADS grades.

Grades	Malignant (Total = 23)	Benign (Total = 77)	*p*-Value
II	0 (0.0%) a	9 (11.7%) a	§ < 0.001 *
III	3 (13.0%) a	62 (80.5%) b	
IV	14 (60.9%) a	6 (7.8%) b	
V	6 (26.1%) a	0 (0.0%) b	

§ Fisher’s exact test. * Significant. Homogenous groups had the same symbol “a,b” based on post hoc Bonferroni test.

**Table 3:** GI-RADS grades among histopathology findings.

Histopathology	GI-RADS				
	II	III	IV	V	Total
Serous cystadenoma	4 (16.7%)	18 (75.0%)	2 (8.3%)	0 (0.0%)	24
Endometrioma	0 (0.0%)	18 (94.7%)	1 (5.3%)	0 (0.0%)	19
Dermoid	0 (0.0%)	11 (78.6%)	3 (21.4%)	0 (0.0%)	14
Mucinous cystadenoma	1 (11.1%)	8 (88.9%)	0 (0.0%)	0 (0.0%)	9
Fibroma	1 (14.3%)	6 (85.7%)	0 (0.0%)	0 (0.0%)	7
Functional cysts	3 (75.0%)	1 (25.0%)	0 (0.0%)	0 (0.0%)	4
Serous cystadenocarcinoma	0 (0.0%)	2 (12.5%)	10 (62.5%)	4 (25.0%)	16
Immature teratoma	0 (0.0%)	0 (0.0%)	3 (75.0%)	1 (25.0%)	4
Mucinous cystadenocarcinoma	0 (0.0%)	1 (33.3%)	1 (33.3%)	1 (33.3%)	3
*p*-value	§ < 0.001 *				

Percentages were obtained from histopathology total (rows). § Fisher’s exact test. * Significant.

**Table 4:** Agreement between GI-RADS diagnosis and final histopathology diagnosis.

GI-RADS	Histopathology	
	Malignant	Benign
IV-V	20 (20.0%) ^TP^	6 (6.0%) ^FP^
II-III	3 (3.0%) ^FN^	71 (71.0%) ^TN^
Kappa (95% CI)		0.757 (0.607–0.907)
*p*-value		<0.001 *

Percentages are from the total (100). TP: True positive. TN: True negative. FP: False positive. FN: False negative. CI: Confidence interval. * Significant.

**Table 5:** Histopathology findings according to GI-RADS accuracy among the studied cases.

GI-RADS Accuracy	Histopathology	*n*	%
True positive (Total = 20)	Serous cystadenocarcinoma	14	70.0%
	Immature teratoma	4	20.0%
	Mucinous cystadenocarcinoma	2	10.0%
True negative (Total = 71)	Serous cystadenoma	22	31.0%
	Endometrioma	18	25.4%
	Dermoid	11	15.5%
	Mucinous cystadenoma	9	12.7%
	Fibroma	7	9.9%
	Functional cysts	4	5.6%
False positive (Total = 6)	Serous cystadenoma	2	33.3%
	Endometrioma	1	16.7%
	Dermoid	3	50.0%
False negative (Total = 3)	Serous cystadenocarcinoma	2	66.7%
	Mucinous cystadenocarcinoma	1	33.3%

**Table 6:** Diagnostic characteristics of GI-RADS in diagnosing malignancy.

Characteristics	Value	95% CI
Sensitivity	87.0%	66.4–97.2%
Specificity	88.6%	83.8–97.1%
Diagnostic accuracy (DA)	91.0%	83.6–95.8%
Youden’s index	79.2%	64.2–94.2%
Positive predictive value (PPV)	76.9%	56.4–91.0%
Negative predictive value (NPV)	92.3%	88.6–99.2%
Positive likelihood ratio (LR+)	11.16	5.09–24.45
Negative likelihood ratio (LR−)	0.14	0.05–0.41
Diagnostic odds ratio (DOR)	78.89	18.10–343.81

CI: Confidence interval.

## Data Availability

Raw data were generated at [Obstetrics & Gynecology Department, Faculty of Medicine, Beni-Suef University]. The data supporting the findings of this study are available from the corresponding author upon request.

## Supplementary Materials

The following supporting information can be downloaded at: https://www.mdpi.com/article/10.3390/medicina61040679/s1, Figure S1: False positive findings among the studied cases; Figure S2: False negative findings among the studied cases.
