# Supplementary material for: Diagnostic Performance of Gynecologic Imaging Reporting and Data System (GI-RADS) in Preoperative Evaluation of Adnexal Masses
Source: Medicina (Kaunas). 2025 Apr 7;61(4):679. doi: 10.3390/medicina61040679 (PMC12028664; doi:10.3390/medicina61040679)
Supplement: Supplementary file 1 [file medicina-61-00679-s001.zip › medicina-3398965-supplementary.pdf]

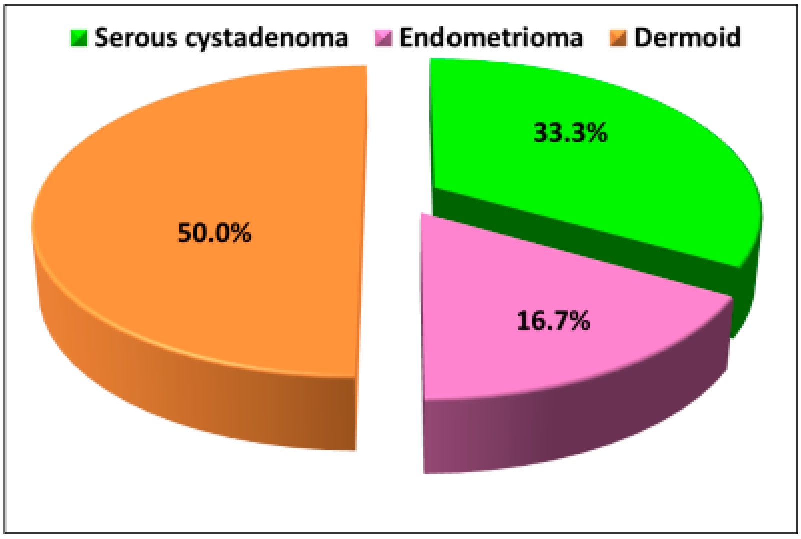

Figure S1. False positive findings among the studied cases.

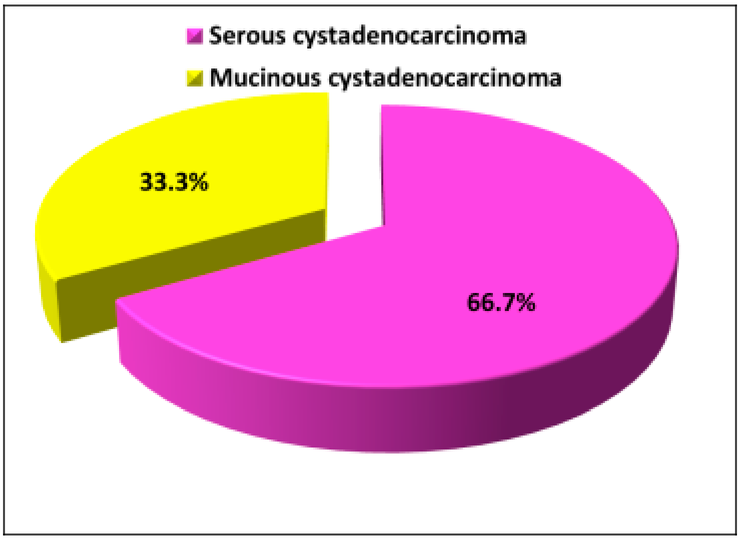

Figure S2. False negative findings among the studied cases.
